# Metabolic Shift of an Isogenic Strain of *Enterococcus faecalis* 14, Deficient in Its Own Bacteriocin Synthesis, as Revealed by a Transcriptomic Analysis

**DOI:** 10.3390/ijms21134653

**Published:** 2020-06-30

**Authors:** Rabia Ladjouzi, Anca Lucau-Danila, Djamel Drider

**Affiliations:** UMR Transfrontalière BioEcoAgro N° 1158, Univ. Lille, INRAE, Univ. Liège, UPJV, YNCREA, Univ. Artois, Univ. Littoral Côte d’Opale, ICV—Institut Charles Viollette, F-59000 Lille, France; rabia.ladjouzi@univ-lille.fr (R.L.); anca.lucau@univ-lille.fr (A.L.-D.)

**Keywords:** biological cost, bacterial physiology, leaderless two-peptides, enterocin EntDD14, biofilm, antimicrobial peptide, microarray analysis

## Abstract

The production of antimicrobial molecules often involves complex biological pathways. This study aimed at understanding the metabolic and physiological networks of enterocin EntDD14-associated function, in the bacteriocinogenic strain, *Enterococcus faecalis* 14. A global and comparative transcriptomic study was carried out on *E. faecalis* 14 and its isogenic mutant Δ*bac*, inactivated in genes coding for EntDD14. The in vitro ability to form biofilm on polystyrene plates was assessed by the crystal violet method, while the cytotoxicity on human colorectal adenocarcinoma Caco-2 cells was determined by the Cell Counting Kit-8. Transcriptomic data revealed that 71 genes were differentially expressed in both strains. As expected, genes coding for EntDD14 were downregulated in the Δ*bac* mutant, whereas the other 69 genes were upregulated. Upregulated genes were associated with phage-related chromosomal islands, biofilm formation capability, resistance to environmental stresses, and metabolic reprogramming. Interestingly, the Δ*bac* mutant showed an improved bacterial growth, a high capacity to form biofilm on inanimate surfaces and a very weak cytotoxicity level. These multiple metabolic rearrangements delineate a new line of defense to counterbalance the loss of EntDD14.

## 1. Introduction

Bacteriocins are proteinaceous ribosomally synthesized antimicrobial peptides that kill or inhibit the growth of undesired bacteria. They have either a narrow spectrum, acting on phylogenetically closely related bacteria [1], or a broad spectrum, acting on phylogenetically distant bacteria [1,2]. Bacteriocins are produced by both Gram-negative and Gram-positive bacteria [3], and to a lesser extent by Archea [4]. Currently there is no unique and universally adopted scheme of bacteriocin classification. Cotter et al. [5] subdivided bacteriocins into two main classes. Class I contains bacteriocins that undergo significant post-translational modifications and class II contains unmodified peptides that only undergo slight modification such as the formation of disulfide bridges or circularization. Due to their cationic nature, and the anionic property the bacterial cell surface, they can form pores in bacterial cell membrane, resulting in dissipation of the proton motive force and depletion of intracellular ATP [6,7]. Bacteriocins can also utilize a set of docking molecules such as lipid II, maltose ABC-transporters, Zn-dependent metallopeptidase, undecaprenyl pyrophosphate phosphatase. For a review, see Cotter et al. [5].

The production of antagonistic substances, such as bacteriocins, is a key (and ancient) mechanism of defense that has been conserved throughout evolution. There have been a large number of bacteriocins isolated from nature. Production of bacteriocins occurs in *Firmicutes*, *Proteobacteria*, *Bacteroidetes*, and *Actinobacteria* according to Drissi et al. [8], who carried out a large genome mining project. Bacteriocins, mainly those produced by lactic acid bacteria, designated as LAB-bacteriocins, have potential applications as food preservatives being a potentially safer alternative to the chemicals used for food preservation [9]. Furthermore, research on bacteriocins has spurred an increasing interest in their other multifaceted biological functions, as reviewed by Drider et al. [10], and Chikindas et al. [11]. They are steadily reported as therapeutic agents to fight malevolent pathogens and drug-resistant bacteria [12,13]. Bacteriocin-encoding DNAs are usually organized into operon clusters on the chromosome or any other DNA genetic element such as plasmids [14]. Their export outside of the producing cell is carried out by two main mechanisms: the ABC-transporter and the sec-dependent pathways [15].

Enterocin DD14 (EntDD14) is a leaderless two peptide bacteriocin produced by *Enterococcus faecalis* 14, a strain isolated from a meconium sample obtained from the Victor Provo Hospital (Roubaix, France) [16]. The strong antibacterial activity of EntDD14 was mainly noticeable against a range of Gram-positive bacteria, including *Staphylococcus aureus*, *Listeria monocytogenes*, *E. faecalis*, *Bacillus subtilis*, and *Clostridium perfringens* [16,17]. The chromosomal genetic organization of the EntDD14 cluster has recently been reported and the isogenic mutant Δ*bac*, inactivated in genes coding for EntDD14 was constructed [18].

The production of bacteriocins is complex and influenced by different environmental factors, such as media composition, pH, or temperature [19], allowing bacteriocinogenic strains to thrive under environmental conditions. Nevertheless, no study so far been reported on the capacity of any bacteriocinogenic strain to reorganize and adapt its metabolism following site-directed inactivation of DNA coding for its bacteriocin. Thus, we establish here through a microarray study, a first snapshot highlighting the physiological and metabolic capabilities of the bacteriocinogenic *E. faecalis* 14 strain to reorganize its cell machinery following the loss of its normal mechanism of defense.

## 2. Results

### 2.1. Gene Expression Analysis in the E. faecalis 14 Δbac Mutant

**Figure 1:**
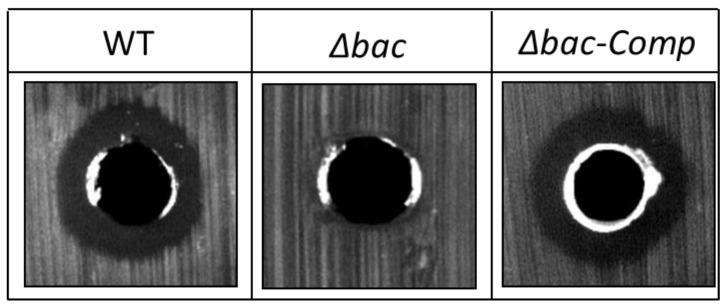
Antimicrobial activity of supernatants of *E. faecalis* 14 wild-type, its isogenic Δ*bac* mutant and Δ*bac-Comp* complemented strains against *L. innocua* at 6 h of growth on GM17 medium. *∆bac: E. faecalis* 14 mutant deleted in *ddA* and *ddB* bacteriocin structural genes. If present, the inhibition zone indicated the susceptibility of the bacterial lawn (*L. innocua*) to the produced EntDD14 bacteriocin. The data are representative of at least three independent experiments.

**Figure 2:**
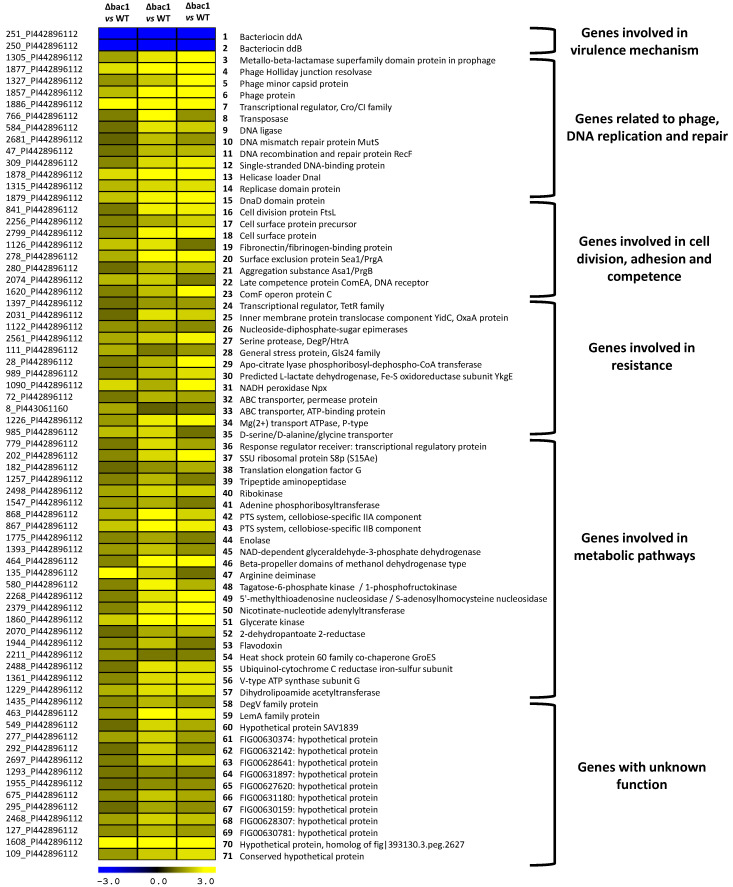
Differentially expressed genes (DEGs) in the *E. faecalis* 14 Δ*bac* mutant strain. DEGs with fold change (FC) >2.0 and <0.5 of individual Δ*bac* (1–3) vs. mean of the wild-type (WT) were represented. The putative functional groups are indicated on the right of the figure.

Previous sequencing of the *E. faecalis* 14 genome showed that the EntDD14 encoding genes are chromosomally located [20]. Recently, we constructed a mutant unable to produce EntDD14 by deleting the *ddA* and *ddB* genes encoding this bacteriocin [18]. To explain the particular behavior of the Δ*bac* mutant strain and the impact of the bacteriocin EntDD14 on the global regulation and gene expression in *E. faecalis* 14, we performed a comparative transcriptomic analysis of RNA isolated from the Δ*bac* mutant strain versus the wild-type (WT), after 6 h of growth in GM17 medium under semi-aerobic conditions. Notably under these conditions, the bacteriocin EntDD14 is produced both in wild-type and in the Δ*bac-Comp* complemented strain but not in the Δ*bac* mutant (Figure 1). The results revealed a total of 71 genes that were differentially expressed in the Δ*bac* strain (Figure 2 and Supplementary Table S1). Of note, only the *ddA* and *ddB* structural genes were as expected downregulated in the mutant Δ*bac* strain (Figure 2, lines 1–2), whereas the 69 other remaining genes were upregulated. The functional assembly indicated that in the absence of a functional bacteriocin gene, numerous other genes were upregulated, enabling most likely a substitutionary induced defensive option (Figure 2).

An important group of genes with phage-related functions was found to be upregulated, including genes coding for a metallo-β-lactamase superfamily domain protein in the prophage, phage Holliday junction resolvase, phage capsid proteins or a bacteriophage transcriptional regulator belonging to the Cro/CI family (Figure 2, lines 3–7). In the same group were embedded genes involved in the transposition or DNA replication and repair (Figure 2, lines 3–15). Genes playing an important role in the cell division, cell adhesion, biofilm formation, or competence mechanisms were also found to be upregulated in *E. faecalis* Δ*bac* mutant strain (Figure 2, lines 16–23). Another group including genes implied in the resistance to antibiotics (transcriptional regulator, TetR family), in the outer envelope biosynthesis (OxaA, Nucleoside-diphosphate-sugar epimerase), in the response to general stress (DegP/HtrA, Gls24 family general stress protein, apo-citrate lyase phosphoribosyl-dephospho-CoA transferase), or more specifically to oxidative stress survival (YkgE, Npx), were also upregulated in the *E. faecalis* Δ*bac* mutant strain, probably related to environmental resistance of this mutant (Figure 2, lines 24–31). In the same group, several transporters acting as efflux pumps were found markedly to be upregulated (Figure 2, lines 32–35). An important set of genes involved in the transcription, translation, and various metabolic pathways such as glycolysis, tagatose pathway, glycerol and arginine catabolism (Figure 2, lines 36–57) were noticed as being upregulated and are likely related to the shift to a new defensive strategy. Finally, a group of 14 genes (Figure 2, lines 58–71) were found to be upregulated in *E. faecalis* Δ*bac* mutant strain but their roles are unknown.

### 2.2. Impact of the Deletion of the Structural Genes Coding for the Bacteriocin on the Bacterial Growth and Ultrastructure

**Figure 3:**
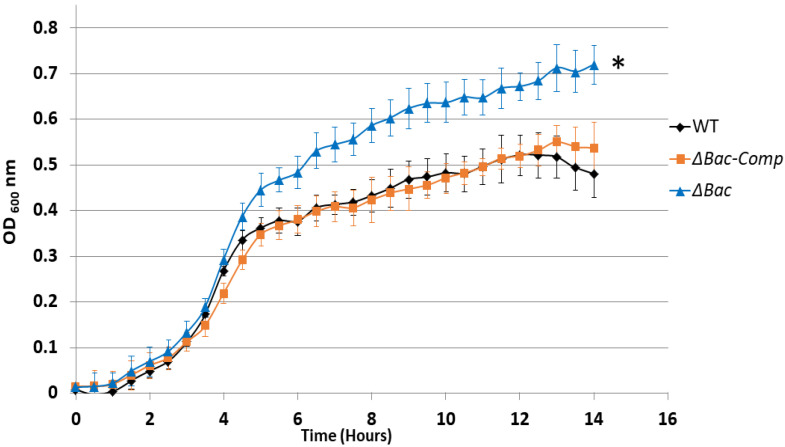
Growth curves of *E. faecalis* strains in GM17 medium. *E. faecalis* 14 WT, Δ*bac* mutant and the Δ*bac* complemented strain. The vertical bars represent the standard deviations. The data are the means of three independent experiments. The asterisk (*) indicates that the growth rate is significantly different from that of the WT strain using the Student *t* test.

As revealed by this transcriptomic analysis, a set of genes involved in metabolism, cell division, transport of molecules, and resistance to oxidative stress were over-expressed in the Δ*bac* mutant as compared to WT. Presumably, these multiple adaptations have developed to counterbalance the absence of bacteriocin EntDD14 synthesis, and could impact on bacterial physiology and growth. To verify this hypothesis, we performed growth kinetics in 96-well microplate in GM17 medium at 37 °C for 14 h. As expected, the Δ*bac* mutant showed better bacterial growth than the WT (Figure 3). The mutant reached a maximum optical density OD_600nm_ of 0.71 which was significantly higher than that of the WT (OD 0.52). The growth rates of the both strains were significantly different (*p* < 0.05). Remarkably, the Δ*bac* strain was able to recover the WT phenotype when transformed by a pAT18 plasmid containing the *ddA* and *ddB* genes which encode a functional bacteriocin EntDD14 as shown by the Δ*bac-Comp* kinetic. Notably, no significant differences were observed between the growth rates of the Δ*bac-Comp* complemented strain and that of the WT (*p* = 0.931), arguing therefore, a role for EntDD14 in the global bacterial growth.

**Figure 4:**
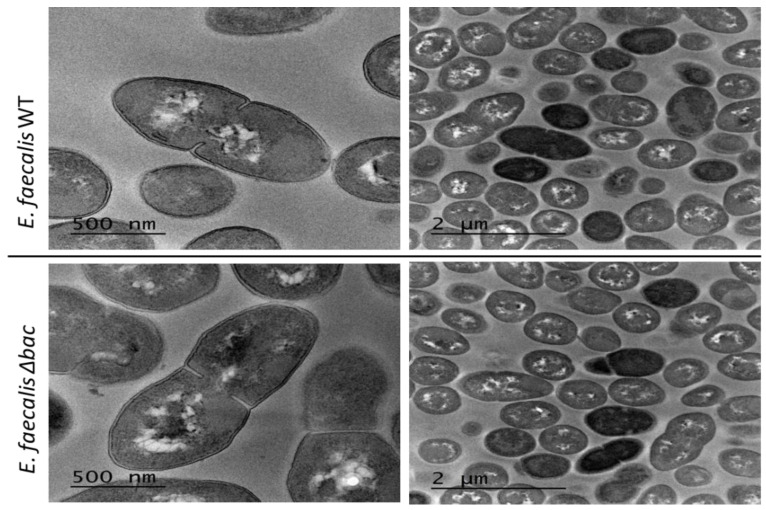
Transmission electron micrographs of *E. faecalis* 14 and its Δ*bac* mutant strain. No significant differences in the size or organization of bacterial cells were observed.

To strengthen the above-presented results, we verified whether any changes in the bacterial cell size, cell shape or other morphological structures (filaments or mucus) can be observed for the Δ*bac* mutant, as opposed to the WT. Such differences might explain the differences in the observed OD, as cell size can directly influence absorbance values. For this reason, exponential phase cultures, after 6 h of growth on GM17 medium under semi-aerobic conditions, were observed by transmission electron microscopy (TEM). Several micrographs of *E. faecalis* 14 and the Δ*bac* mutant were compared but no significant differences were noticed in the cell ultrastructure (Figure 4). Both strains exhibited cells of similar size and morphology of the bacterial cell wall with a predominant diplococcal organization. It was noted that both strains displayed colonies of the same appearance and average size when cultured on GM17 agar.

### 2.3. Absence of EntDD14 and the Biofilm Production Ability of the Δbac Mutant Strain

**Figure 5:**
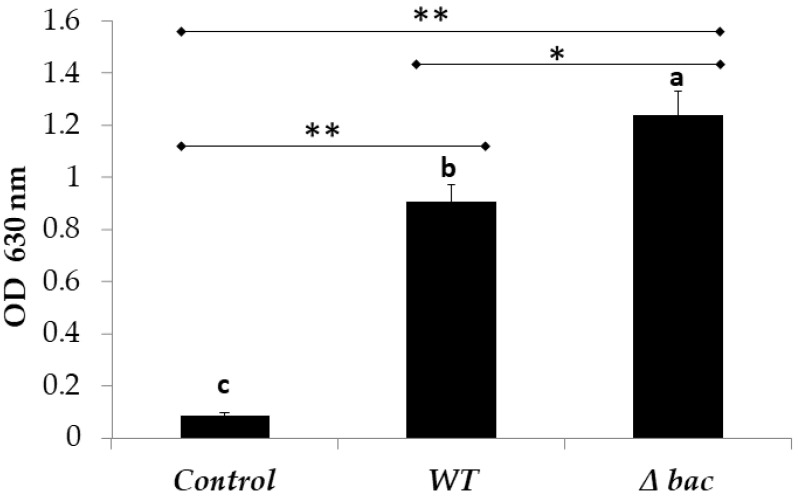
Adhesion of *E. faecalis* 14 and its Δ*bac* mutant strain to polystyrene plates as determined by OD_630nm_ measurements. The absorbance values are the means of three independent experiments. Sterile GM17 was used as control. The error bars represent the standard deviations. Columns with different letters are significantly different using one-way ANOVA with Tukey test and Student *t* test for pairwise comparisons. (*), *p* < 0.05; (**), *p* < 0.01.

Among the genes over-expressed in the Δ*bac* mutant, two groups were related to biofilm formation and maintenance. Therefore, we assessed the ability of the Δ*bac* mutant and WT strains to form biofilm using crystal violet method on polyester plates. A deeper analysis revealed a better adhesion score for the Δ*bac* mutant compared to the WT (Figure 5). There were significantly higher absorbance values recorded for the Δ*bac* mutant (OD_630nm_ = 1.241) compared to WT (OD_630 nm_ = 0.909) with a *p* value of *p* = 0.011. Nevertheless, following the recommendations of Stepanović et al. [21] we considered both strains to be strongly adherent, since the recorded absorbance values were 4 times greater than that of the control (OD_630nm_ = 0.09). These results confirmed those obtained by transcriptomic analyses and showed that a mutant deleted in the genes coding for bacteriocin increased its adhesion capacity and consequently its biofilm formation.

### 2.4. Absence of Bacteriocin Reduced the Cytotoxicity of E. faecalis 14

**Figure 6:**
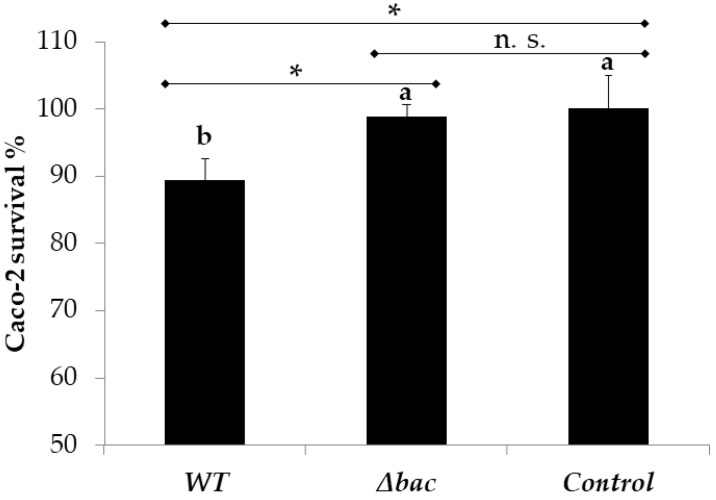
Cytotoxicity of *E. faecalis* 14 and its Δ*bac* mutant on Caco-2 cells (24 h). Data are means of three independent experiments. Error bars represent standard deviations. Columns with different letters are significantly different using one-way ANOVA with Tukey test and the Student *t* test for pairwise comparisons. (*), *p* < 0.05; (**), *p* < 0.01; not significant (n.s.), *p* > 0.05.

We previously showed that neither *E. faecalis* 14 nor its enterocin EntDD14 were cytotoxic to the intestinal porcine epithelial cell line IPEC-1 [17]. Here, we tested the cytotoxicity of *E. faecalis* 14 against Caco-2 cells. As expected, *E. faecalis* 14 exert only a slight cytotoxic effect on these eukaryotic cells, since 89% survival was observed after 24 h of contact (Figure 6). Interestingly, in the absence of EntDD14 bacteriocin the viability of Caco-2 cells increased after 24 h incubation, as shown by a very weak cytotoxicity level of the Δ*bac* mutant on Caco-2 cells (viability of 98.8%, Figure 5). The difference between the Δ*bac* mutant and WT was significant but with a *p* value close to the 0.05 cut-off value (*p* = 0.045).

## 3. Discussion

Bacteriocins are known for their effectiveness in fighting and eradicating microbial pathogens including bacteria, and to a lesser extent fungi and viruses [22,23,24]. Initially, the bacteriocin synthesis and its impact on a specific bacteriocin gene was studied at the transcriptional level, and in very limited cases at the post-transcriptional levels. To the best of our knowledge, there are no data reporting the impact of bacteriocin on the whole bacterial transcriptome. Thus, there is a need to understand how a bacteriocinogenic strain or any probiotic strain would behave when it is naturally or purposely deprived of the ability to produce bacteriocin. In fact, this would happen following a spontaneous mutation of one or more genes involved in the biosynthesis of the bacteriocin. To gain new insights into this important question we conducted a comparative transcriptomic study using the total RNA isolated from the bacteriocinogenic *E. faecalis* 14 and the Δ*bac* mutant strain, after 6 h of growth. The Δ*bac* mutant, previously engineered to delete its structural genes encoding the peptides A and B of the EntDD14 bacteriocin, was unable to produce this bacteriocin [18].

Overall, the loss of EntDD14 synthesis caused metabolic and physiological changes in *E. faecalis* 14 to result in what is probably an alternative mechanism of defense. Thus, genes involved in mobile genetic element activity and biofilm formation were upregulated in the Δ*bac* mutant, as well as genes involved in resistance to environmental stresses and metabolism, thereby proving the ability of this strain to adapt and thrive under the experimental conditions tested here. These transcriptomics data were consolidated by the assays of adhesion to polystyrene plates, and the assessment of cytotoxicity to Caco-2 cells. However, deeper analyses using additional experiments such as a qRT-PCR or transcriptional fusions are needed to strengthen these conclusions.

**Figure 7:**
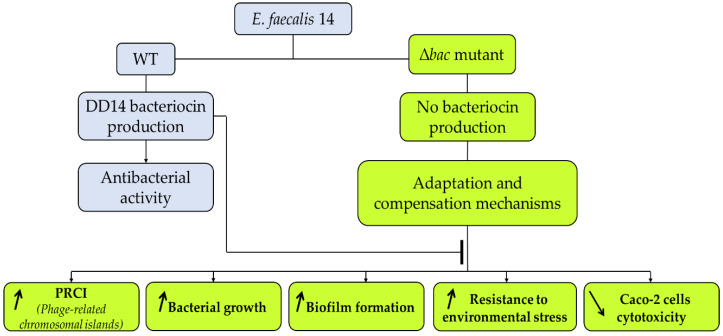
Model proposing the impact of deletion of genes encoding EntDD14 bacteriocin in *E. faecalis* 14.

The production of bacteriocins might provide a competitive advantage to a producer in certain ecological niches [25]. This would explain the high frequencies of bacteriocin-producing enterococci in the human intestinal tract [26]. So, in the absence of this key element, the bacterium is able to reorganize its metabolism, to provide an alternative mechanism to maintain its competitiveness in the environment (Figure 7).

Transcriptomic data obtained here show that *E. faecalis* can enhance expression of several genes in order to compensate for the loss of the antibacterial activity attributable to EntDD14.

The group of DEGs involved in the phage activity and also in DNA replication and repair (Figure 2, lines 3–15) includes genes that present high homology with prophage-related genes described in *E. faecalis* V538 [27] and obvious functional similarities with the phage-related chromosomal islands (PRCIs) that were first identified in *Staphylococcus aureus* [28]. Closely related elements with the same genetic organization were reported as well in *E. faecalis* PRCIs [28], and mobile genetic elements were found to act as a reservoir for acquisition and dissemination of drug resistance factors in this species [29]. Matos et al., showed that phage-related genes play an important role in genetic and physiological flexibility for optimal adaptation in *E. faecalis* V583 [27]. These authors indicated that prophage induction in *E. faecalis* subpopulations could favor their survival and found this mechanism to be similar to that described for *Shewanella oneidensis*, *Pseudomonas aeruginosa* and *Streptococcus pneumoniae*, especially in biofilms [30,31,32].

Besides these putative PRCI, other genes involved in the cell division, cell adhesion, biofilm formation, and competence were found to be upregulated in the Δ*bac* mutant (Figure 2, lines 16–23). Interestingly, genes with similar functions were reported as involved in *Enterococcus* biofilm formation [33]. FtsL is an essential protein for the cell division [34] and it was found to be involved in *E. faecalis* biofilm formation [35]. Different cell surface proteins and aggregation substances were reported to be involved in *E. faecalis* biofilm maintenance [36]. The late competence protein ComEA or the ComF operon protein C are involved in competence, a function that in *Streptococcus* mutants is required for biofilm formation [37]. Importantly, our results suggest that the biofilm generation was stimulated in the absence of the bacteriocin active gene in *E. faecalis.* The biofilm formation was already described as the basis of antibiotic resistance in *E. faecalis* [35], and also as a factor in resistance to environmental stress [38]. In this study, we provide a new insight and show that the bacteriocinogenic *E. faecalis* 14, genetically modified and thus deprived of its bacteriocin production, has increased its biofilm formation ability (Figure 5). Interestingly, this form of cellular organization can enhance the bacterial persistence and resistance to environmental stress [39,40,41], which correlates with our own observations (Figure 2, lines 24–35). Moreover, the involvement of enzymes such as NADH peroxidase Npx, lactate dehydrogenase as well as the general stress protein Gls24, in the response to oxidative stress, has already been demonstrated in *E. faecalis* [42,43,44]. Of note, the expression of genes coding for these enzymes in the Δ*bac* mutant indicates a better ability to maintain its redox balance compared to WT. Such adaptation enabled the strain to withstand different environmental stresses, and tolerance to antibiotics, which is correlated with the resistance to oxidative stress [45,46,47].

The lack of bacteriocin and enhancement of other putative virulence mechanisms caused this strain to undergo metabolic reorganization. In this respect, different mechanisms have been described in bacteria to explain the relationship between biofilm formation and the metabolic reprogramming [41,48,49]. This would probably explain the elevated in vitro growth rate observed in the Δ*bac* mutant, which is accompanied by overexpression of genes involved in the glycolysis, tagatose, glycerol, and arginine pathways (Figure 2, lines 36–57).

Furthermore, the tests on Caco-2 cells revealed a very weak cytotoxicity for the Δ*bac* mutant which would therefore presumably have little impact on intestinal cells. Nevertheless, this finding does show indirectly that bacteriocin EntDD14 can produce a very low level of cytotoxicity. A previous study showed no reduction in cell viability at concentrations of 50 μg/mL and 100 μg/mL of pure EntDD14 after 4 h of contact with the intestinal epithelial cell line IPEC-1. However, a decrease of 9.6% to 20% in the IPEC-1 cell viability was observed after 24 h of contact in a dose-dependent manner [17].

Collectively these results revealed alternative compensatory mechanisms present in the Δ*bac* mutant which counterbalance the loss of EntDD14 synthesis and could help the bacterium to maintain its competitiveness in complex environments such as the intestinal tract. This work will open new prospects for studying and better understanding the bacteriocin networks and mainly its role in bacterial pathogenicity.

## 4. Materials and Methods

### 4.1. Bacterial Strains and Growth Conditions

*E. faecalis* 14 [16] and the recently constructed *E. faecalis* 14 Δ*bac* mutant and *E. faecalis* 14 Δ*bac*-*Comp* [18] were used in this study. The Δ*bac* mutant deleted in *ddA* and *ddB* genes was constructed by allelic exchange using a method based on the conditional replication of the pLT06 vector [50], while the complemented strain was constructed with a DNA fragment containing the entire *ddA*, *ddB* and the promoter region cloned in the Gram-positive replicative plasmid pAT18 [51]. Cultures were grown on M17 medium supplemented with 0.5% (*w*/*v*) of glucose (GM17) at 37 °C under semi-aerobic conditions. Growth kinetics were determined in 96-well microplates by measuring the optical density at 600 nm (OD_600_) using a SpectraMax i3 spectrophotometer (Molecular Devices, San Jose, CA, USA). The wells were equally inoculated, and the plates were read every 30 min for 12 h. The bacterial master cultures were stored at −80 °C in GM17 broth supplemented with 50% (*v*/*v*) glycerol.

### 4.2. Antibacterial Activity Assays

The screening of antimicrobial activity of WT *Ent. faecalis* 14, its isogenic Δ*bac* mutant and the complemented Δ*bac*-*Comp* strains against *L. innocua* ATCC 33090 [52] was performed using well-diffusion method [17]. Briefly, Brain Heart Infusion (BHI) plates (1% agar) were inoculated with *L. innocua* strain and were allowed to dry. Then, 50 μL of culture supernatant of the tested strain was added to the well and incubated overnight at 37 °C. The radius of the inhibition zone was measured from the edge of the well to the edge of the inhibition halo.

### 4.3. RNA Isolation and Microarrays Analysis

For the microarray analysis, three distinct cultures of *E. faecalis* 14 Δ*bac* were used (*Δbac1*, *Δbac2* and *Δbac3*) which were compared with *E. faecalis* 14 (WT1, WT2 and WT3), after 6 h of growth in GM17 medium. All cultures were harvested at the same cell concentration of ~2.10^8^ CFU/mL. Analyses were performed with total RNA isolated by using NucleoSpinTM RNA Plus columns (Macherey-Nagel, Hoerdt, France). RNA quality was determined by Nanodrop and the absorbance ratios A260/280 and A260/230 were found to be between 2.0 and 2.2. RNA quality was also examined with a Bioanalyzer 2100 (Agilent, Les Ulis, France) and a minimal RNA integrity number (RIN) of 0.8 was required for all samples.

Agilent G2509F *E. faecalis* 14 custom oligo-based DNA microarray (8 × 15 K) containing spots of 60-mer oligonucleotide probes (in 5 replicates) were used to study the gene expression. RNA amplification, staining, hybridization, and washing were conducted according to the manufacturer’s instructions. Slides were scanned at 5 µm/pixel resolution using the GenePix 4000B scanner (Molecular Devices Corporation, Sunnyvale, CA, USA). Images were used for grid alignment and expression data digitization using GenePix Pro 6.0 software. Expression data were normalized by Quantile algorithm. To ascertain the quality of normalized data, filtering of data was mandatory for flagged signals. Expression data of the 3 wild-type samples were filtered for *p* value < 0.05 and the average was calculated for each gene. A fold change (FC) value was calculated using Δ*bac* individual samples and the mean of WT. Differentially expressed genes (DEGs) were selected for a FC threshold >2.0 or <0.5 and transformed in log2 FC. As results of biological repetitions were significantly similar, no qRT-PCR validation was performed. Functional annotation of DEGs was based on NCBI GenBank and related-genes physiological processes were assigned with NCBI, AmiGO 2 Gene Ontology and UniProt. KEGG pathway analysis was also used to identify relevant biological pathways of selected genes. All the microarray data have been submitted to the NCBI GEO archive for functional genomics data with the accession number GSE149873.

### 4.4. Transmission Electron Microscopy

Cultures of *E. faecalis* 14 and its Δ*bac* derivative mutant were grown in GM17 at 37 °C for 6 h then harvested by centrifugation (×8000 *g*, 10 min, 4 °C). For TEM, the pellets were fixed with 2.5% (*v*/*v*) glutaraldehyde solution and 0.1 M (*v*/*v*) of Cacodylate buffer (pH 7.4) and prepared as a Formvar film on a 300 square mesh, nickel grid (EMS FF300-Ni). The TEM images were obtained using a JEOL JEM 2100FX TEM instrument (Jeol, Tokyo, Japan) equipped with a GATAN CCD Orius 200D camera (Gatan, Pleasanton, CA, USA) at an acceleration voltage of 200 KV.

### 4.5. Assessment of Biofilm Formation by Enterococcal Strains on Polystyrene Tissue Culture Plates (TCP)

To assess the biofilm formation of *E. faecalis* 14 and its Δ*bac* derivative mutant strains to polystyrene plate, a semi quantitative method was used, as previously described [53]. Briefly, 100 μL of culture of each *Enterococcus* strain (10^8^ CFU/mL), grown in GM17, were added to the wells of sterile 96-well microplates already filled with 100 μL of sterile GM17. The microplates were left for 15 min with gentle agitation before being incubated at 37°C for 24 h. The cultures were then aspirated and the non-adherent cells were removed by two washes with phosphate-buffered saline (PBS) 10 mM, pH 7.2. Subsequently, 200 μL of 96% ethanol (Sigma–Aldrich, St Louis, MO, USA) were added to each well in order to fix the adherent cells. After 15 min of fixation, the wells were drained, dried and then stained with 0.1% (*w*/*v*) crystal violet (Biochem Chemopharma, Quebec, Canada) for 30 min. The stained cells were washed twice with 200 μL of PBS before extracting the dye with 200 μL of 96% ethanol. The number of cells was quantified using a microplate reader (ELX800. BioTek, Winooski, VT, USA) by measuring the absorbance (A) at 630 nm. According to the recommendations of Stepanović et al. [21], these strains were classified into four categories. Taking Ac as the absorbance of the control (sterile GM17), the following interpretations were applied; A ≤ Ac: non-adherent (non-biofilm producer), 2Ac ≥ A > Ac: weakly adherent (weak biofilm producer), 4Ac ≥ A > 2Ac: moderately adherent (moderate biofilm producer), and strongly adherent (strong biofilm producer): A > 4Ac.

### 4.6. CCK-8 Cytotoxicity Assay

Cytotoxicity of *E. faecalis* 14 wild-type and its Δ*bac* derivative mutant strains was assessed in vitro using the Cell Counting Kit-8 (CCK-8) assay (Dojindo Molecular Technology, Kumamoto, Japan), for Caco-2 cells [54]. Caco-2 cells were seeded at a density of 6 × 10^4^ cells/well in 96-well cell culture plates and preincubated for 7 days at 37 °C in the presence of 5% CO_2_ in Dulbeco’s Modified Eagle Medium (DMEM) (Thermo Fisher Scientific, Courtaboeuf, France) containing 4.5 g/L of glucose and supplemented with L-glutamine (2 mM), penicillin (100 U/mL), streptomycin (100 µg/mL), 10% of heat-inactivated fetal bovine serum (FBS) and 1% (*v*/*v*) non-essential amino acids. All these reagents were provided by PAN-Biotech GmbH (Aidenbach, Germany). Of note; the media were changed three times to maintain optimal conditions for Caco-2 cell growth. Overnight enterococcal cultures were prepared in DMEM without antibiotics and then applied to confluent Caco-2 cell monolayers at a ratio of MOI (Multiplicity of infection) 1:10 (Caco-2/*E. faecalis* 14 or Δ*bac* mutant strains). A control test was also performed with non-infected Caco-2 cells. After 24 h of incubation, the medium was removed, and cells were washed twice with PBS and then incubated with 150 μL of DMEM containing gentamicin (50 μg/mL) and 5% of CCK-8 reagent for 2 h at 37 °C. The relative viability (%) was then calculated based on absorbance at 450 nm using a microplate reader (Xenius SAFAS, Monaco, France). Results were expressed as percentage of proliferation compared to the viability of untreated cells (control).

### 4.7. Statistical Analysis

Differences in absorbance values among samples were calculated using the Student *t* test. *p* values of less than 0.05 were considered to be significant. Data of bacterial growth, biofilm formation, and cytotoxicity experiments were expressed as a mean ± standard error calculated over three independent experiments. Analysis of statistical significance was performed by one-way ANOVA and the post-hoc Tukey Test (*p* < 0.05).

## 5. Conclusions

This paper reports for the first time a comparative transcriptomics analysis of a bacteriocinogenic strain, namely *E. faecalis* 14 and its derivative strain, inactivated in genes coding for synthesis of its own bacteriocin EntDD14 Δ*bac*. The mutant strain Δ*bac* could express differentially at least 71 genes. Two out of 71 genes were downregulated and 69 out of 71 were upregulated. Downregulated genes were those coding for EntDD14 because these genes had been removed from this strain. Upregulated genes included those coding for proteins involved in cellular defense and virulence, probably as a substitutionary line of defense. These include a group that could be associated with PRCIs, probably involved in physiological flexibility and optimal adaptation. Other upregulated genes were associated with cell division, cell adhesion, and biofilm formation. Genes involved in the environmental resistance were markedly upregulated and a prominent metabolic reprogramming accompanied all these strategies. In addition to these transcriptomic observations, the Δ*bac* mutant possessed better growth, a high capacity to form biofilm on inert surfaces and a very weak level of cytotoxicity on Caco-2 cells. Collectively, these transcriptomic data suggest a new alternative virulence strategy in case of inability to synthesize bacteriocin.

## Supplementary Materials

Supplementary materials can be found at https://www.mdpi.com/1422-0067/21/13/4653/s1, Table S1: Gene expression in *Enterococcus faecalis* 14 Δ*bac* mutant strain.
